# Tar yield and composition from poultry litter gasification in a fluidised bed reactor: effects of equivalence ratio, temperature and limestone addition†

**DOI:** 10.1039/c9ra02548k

**Published:** 2019-04-30

**Authors:** Alen Horvat, Daya Shankar Pandey, Marzena Kwapinska, Barbara B. Mello, Alberto Gómez-Barea, Lydia E. Fryda, Luc P. L. M. Rabou, Witold Kwapinski, James J. Leahy

**Affiliations:** Carbolea Research Group, Department of Chemical Sciences, Bernal Institute, University of Limerick Limerick V94 T9PX Ireland alenhorvat@hotmail.com Alen.Horvat@ul.ie; School of Engineering and the Built Environment, Anglia Ruskin University Chelmsford Essex CM1 1SQ UK; Technology Centre for Biorefining & Biofuels, University of Limerick Limerick V94 T9PX Ireland; Chemical and Environmental Engineering Department, Escuela Técnica Superior de Ingeniería, University of Seville Camino de los Descubrimientos s/n 41092 Seville Spain; Energy Research Centre of the Netherlands (ECN), Biomass & Energy Efficiency Petten The Netherlands

## Abstract

Air gasification of poultry litter was experimentally investigated in a laboratory scale bubbling fluidised bed gasifier. Gasification tests were conducted at atmospheric pressure using silica sand as the bed material. This paper examines the effect of the equivalence ratio (ER) in the range of 0.18–0.41, temperature between 700 and 800 °C, and the addition of limestone blended with the poultry litter on the yield and composition of tar. An off-line solid phase adsorption method was employed in order to quantify tar compounds heavier than styrene, whereas lighter species such as benzene and toluene were measured by means of on-line micro gas chromatography. Total tar yields were in the range from 15.7 to 30.7 g_total tar_ kg_poultry litter (dry and ash free basis)_^−1^. These values are considered low with respect to the feedstocks with a higher organic fraction. It also needs to be noted that the yields of benzene and toluene were measured by on-line micro gas chromatography, a technique which inherently delivers higher tar values compared to commonly employed off-line techniques. By varying the ER, poultry litter blended with limestone showed a reduction in total tar yield whereas poultry litter on its own showed an increasing tar yield over the ER range tested. In the presence of limestone, polycyclic aromatic hydrocarbons (PAHs), heterocyclic compounds, toluene and benzene showed a tendency to reduce over the ER range tested. Since the ER also plays a crucial role in tar reduction, the reduction in tar cannot be unambiguously attributed to calcined limestone/lime (CaCO_3_/CaO). Increasing the temperature was shown to be effective for reducing the total tar yield but the amounts of polycyclic aromatic hydrocarbons increased. However, no definitive correlation could be established between limestone/lime catalytic activity for tar reduction and elevated gasification temperature, because there was no possibility to study their effects separately. The chemical composition of the tar arising from poultry litter is distinctive compared with conventional lignocellulosic fuels linked to the fact that poultry litter has a higher nitrogen content (≈6.5% w/w (dry and ash free basis)). Nitrogen-containing hydrocarbons such as pyridine, 2-methylpyridine, 2-methyl-1*H*-pyrrole and benzonitrile were identified in significant amounts. This study has demonstrated that poultry litter gasified in a bubbling fluidised bed yielded a product gas with relatively low tar content while its composition reflects the chemical nature of the feedstock.

## Abstract 2

Qualitative and quantitative measurements of tar from poultry litter gasification in an air-blown fluidised bed.

## Graphical abstract

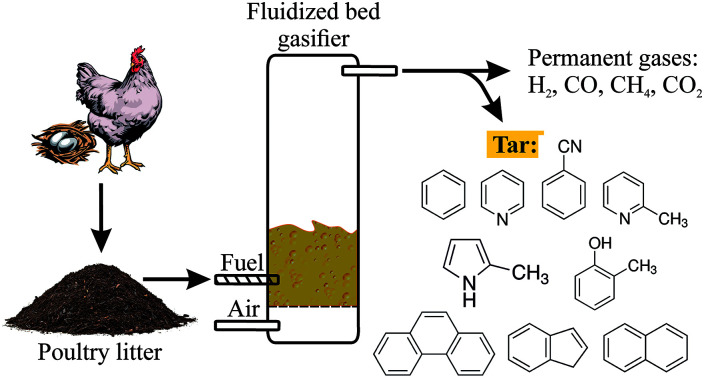

## 1. Introduction

According to the AVEC annual report 2016,^1^ the European Union is the world's leading supplier of poultry meat with an annual production of 13.6 million tonnes in 2015. The report also predicted a growth rate of about 1% a year. Even though intensive livestock production has become a more economically viable option compared to traditional farming practices, such industrialised production faces issues associated with its environmental impact due to the accumulation of large quantities of waste with estimates of 1.4 billion tonnes per annum^2^ of manure in EU states. The increasing popularity of free range and organic farming supported by European Directives 2007/43/EC and 1999/74/EC requires poultry farmers to comply with minimum animal welfare standards which results in an increased volume of poultry litter due to utilisation of bedding material (*i.e.* wood shavings, straw and hay). Poultry litter is a heterogeneous fuel composed of bedding material, excreta, waste feed and feathers. Compared to conventional lignocellulosic feedstocks, poultry litter is recognised as a low value fuel due to its relatively high moisture and ash content. It is also a source of nutrients such as nitrogen, phosphorous and potassium.^3^

The recent European Commission (EU) regulation no. 592/2014 paves the path for combustion of poultry litter and on-farm utilisation of the energy. Combustion remains the most advanced and widely applied technology with several commercial scale incinerators of poultry litter currently being used for electricity generation and ash recovery in the UK, the USA and The Netherlands.^4^ Recently poultry litter has been successfully gasified in a fluidised bed gasifier^5,6^ as well as in a fixed/moving bed gasifier.^7^ These studies have concluded that due to the high content of elements such as phosphorous and potassium, poultry litter is prone to provoke sintering and agglomeration when gasified in a fluidised bed gasifier. To avoid these problems limestone/lime (CaCO_3_/CaO) have been added to the bed during industrial scale fluidised bed combustion^8^ and laboratory scale fluidised bed gasification^6^ of poultry litter.

Tar is a by-product of thermal gasification processes defined as a generic (unspecific) term accounting for all organic compounds in the product gas excluding benzene and lighter gaseous hydrocarbons.^9^ Although CEN/TS 15439, 2006 ^9^ provides a standardised definition of tar, in practice tar is defined by restrictions of sampling, the identification and quantification methods applied as well as the final application of the product gas or syngas. For example, the solid phase adsorption (SPA) sampling method combined with gas chromatography (GC) detection provides accurate results for phenolic and 2–5 rings polycyclic aromatic tar compounds. However, measurement reliability drops remarkably when either light volatile compounds (*i.e.* benzene) or 6 + ring polycyclic aromatic tar compounds are to be quantified. Tar is a black viscous material potentially giving rise to system malfunction if condensation occurs. In practice, tar needs to be removed for applications other than direct burning of the product gas.^10^ Tar from poultry litter gasification in a fluidised bed reactor has not been reported to date. Although, it has been established that a large portion of the nitrogen present in poultry litter is converted into NH_3_ and HCN during fluidised bed gasification^6^ it is expected that the high nitrogen content in poultry litter would also deliver a wide variety of nitrogen-containing compounds in the tar. Jaramillo-Arango *et al.*^11^ investigated the composition of pyrolysis oil from fluidised bed tests employing nitrogen rich sewage sludge. They found notable amounts of aliphatic acetamide, single aromatic ring species such as pyridine, pyrimidine, pyrrole, aniline and benzonitrile as well as two ring structures such as quinoline and indole. The composition of the tar from poultry litter gasification is expected to reflect the high nitrogen and low lignin content in the feedstock. The formation and decomposition of poultry litter tar is further discussed in the Section 3.

It is well known that tar can be decomposed catalytically with limestone/lime which is an inexpensive, abundant and naturally occurring non-toxic material.^12,13^ Additionally, it has been reported that Ca-based additives enhance pyrolysis conversion rates.^14^ An activation energy of 46 kJ mol^−1^ for tar cracking over a calcined dolomite (CaO–MgO) and 77 kJ mol^−1^ over an inert bed of silica sand was reported by Delgado *et al.*^15^ In regards to tar mitigation, Simell *et al.*^12^ tested the catalytic activity of carbonate rocks by passing model tar compounds over a fixed catalytic bed. The authors concluded that calcined CaO shows good catalytic activity towards tar reduction/reforming. In contrast, a much slower reduction rate has been observed for raw CaCO_3_, which is so slow that it is considered not to occur. However, CaO converts into the carbonated form CaCO_3_, when the CO_2_ partial pressure is higher than that of the equilibrium pressure at the process temperature. Tests have shown that at 900 °C CaO was carbonated to CaCO_3_ only if the partial pressure of CO_2_ was higher than 100 kPa. On the other hand, Valverde and Medina^16^ demonstrated that the calcination reaction triggers at 857 °C under the 50% CO_2_ atmosphere. Rapid calcination has also been observed at temperatures below 857 °C. With respect to gasification the CO_2_ yield is unlikely to reach the equilibrium partial pressure that could be able to reverse the calcination process. Bedyk *et al.*^17^ found out that in inert argon atmosphere CO_2_ is released from CaCO_3_ in the temperature range 500–700 °C forming CaO. While tests in a pure CO_2_ atmosphere and a temperature range of 600–800 °C resulted in CO_2_ adsorption forming CaCO_3_. In the same CO_2_ atmosphere CO_2_ release occurred at the temperatures above 850 °C.

Saw and Pang^13^ tested the extent of tar reduction with 0%, 50% and 100% lime as the bed material. The total tar concentration (sum of all tar compounds) decreased exponentially from 5.0 to 0.7 g Nm^−3^ as the lime loading increased from 0% to 100%. A significant reduction was also observed for all the individual tar compounds studied. Tar reduction with lime loading is most likely due to the steam reforming of tar in the presence of CaO. The steam reforming reactions of the phenol, cresols and toluene are shown in eqn (1)–(4).

1 C_6_H_5_OH + 5H_2_O ↔ 6CO + 8H_2_

2 (CH_3_)C_6_H_4_OH + 13H_2_O ↔ 7CO_2_ + 17H_2_

3 C_7_H_8_ + 7H_2_O ↔ 7CO + 11H_2_

4 C_7_H_8_ + 14H_2_O ↔ 7CO_2_ + 18H_2_

Enhanced production of H_2_ may have a negative effect on the tar steam reforming rate because H_2_ decreases the catalytic activity of CaO due to the adsorption of H_2_ onto its active sites, diminishing the available sites for tar adsorption.^13^ Likewise CaO can react with other gasification products (*i.e.* H_2_O, H_2_S and char/coke) that may deteriorate its catalytic activity for tar reduction.^15,17–19^

In this paper, an experimental study to characterise the yield and composition of tar in the gas during poultry litter gasification in a laboratory scale fluidised bed reactor is presented. The objectives of this study are to investigate the effect of (a) equivalence ratio (ER), (b) limestone addition (blended with the poultry litter) and (c) reactor temperature, on tar yield and its composition. Some basic data regarding overall tar yields were published by Pandey *et al.*,^6^ in a complementary publication to the present work. However, a detailed tar analysis is presented here, including the change of tar composition with the process variables. An additional key aspect of the present work is a discussion on the evolution of nitrogen-containing tar compounds present in the product gas.

## 2. Materials and methods

### 2.1 Materials

Detailed description of poultry litter collection, preparation and characterisation including detailed chemical composition of the poultry litter ash can be found elsewhere.^6^ However, a summary of relevant information is presented here. The bulk density of the partially dried poultry litter was 360 kg m^−3^, with a particle size between 0.7 and 2.8 mm. The limestone used in this study was supplied by Rheinkalk GmbH (Brilon, Germany) with a particle size in the range of 0.9–1.2 mm. Ultimate and proximate properties, chemical composition as well as heating value of the poultry litter are reported in Table 1. The content of fixed carbon was calculated by subtracting the moisture, ash and volatile matter content from 100%. Likewise, the oxygen content in the fuel was calculated by difference.

**Table 1:** Chemical characteristic of poultry litter^6^

**Proximate analysis (% w/w)**	
Moisture (a.r.)	22.10
Volatile matter (d.b.)	73.65 ± 0.02
Ash (d.b.)	17.55 ± 0.06
Fixed carbon a (d.b.)	8.81 ± 0.02
LHV (MJ kg^−1^) (a.r.)	13.53 ± 0.41

**Ultimate analysis (d.a.f.) (% w/w)**	
N	6.48 ± 0.01
C	54.70 ± 0.37
H	6.43 ± 0.07
S	0.90 ± 0.03
Cl	0.70 ± 0.02
O^a^	30.79 ± 0.25

**Chemical composition (d.b.) (wt%)**	
Hemicellulose	11.72
Cellulose	12.88
Lignin	14.16
Extractives^b^	39.21

a Calculated by difference, a.r. – as received, d.b. – dry basis, d.a.f. – dry and ash free basis.

b Containing water and ethanol extractives.

### 2.2 Experimental facility

The gasification experiments were conducted using a laboratory scale air-blown bubbling fluidised bed gasifier located at the Energy Research Centre of The Netherlands (ECN). Experiments were performed at different temperatures (700, 750 and 800 °C) and at different ERs between 0.18 and 0.41 by adjusting the air and N_2_ flow rate while maintaining the total flow at 12 dm^3^ min^−1^ (Table 2). Along with that an effort has been made to maintain a constant feedstock feed rate. The downstream sections of the reactor up to the cold filter were insulated and maintained at 400 °C in order to avoid tar condensation. Tar samples were taken through a SPA sampling port located after the hot filter. Silica sand with a particle size between 0.25 and 0.50 mm (mean particle size of 0.31 mm) and bulk and absolute densities of 1422 and 2620 kg m^−3^ respectively was used as the bed material. To avoid any influence of accumulated ash from previous experiments and to reduce the risk of bed agglomeration, 1.2 kg of fresh silica sand was introduced at the beginning of each experimental day. Gasification experiments were conducted in such a way that the fluidising regime remained constant throughout the tests. The minimum fluidising velocity was around 0.097 m s^−1^ at 20 °C, calculated according to Wen and Yu's correlation.^20^ Each test undertaken at the specified gasification conditions lasted about an hour. Steady state was usually reached within the first 30 min after commencing fuel feeding. The final 30 min were dedicated to the tar sampling and analysis of permanent gases using an on-line micro gas chromatograph (μGC) (Varian, CP-4900). Relevant information comprising schematic diagram of gasifier, technical data and operating conditions of the experimental setup was previously presented by Pandey *et al.*^6^ and are also concisely outlined in Table 2. It is worth mentioning that tests 1, 2 and 3 were carried out using only poultry litter, while tests from 5 to 14 were conducted with poultry litter blended with 8% w/w of limestone.

**Table 2:** Summary of operating conditions^a^ during the fluidised bed gasification of poultry litter^6^

Test number	1	2	3	5	6	7	9	10	11	13	14
Feedstock type	Poultry litter			Poultry litter with 8% w/w limestone			Poultry litter with 8% w/w limestone			Poultry litter with 8% w/w limestone	
Poultry litter feed rate, kg h^−1^ (a.r.)	0.66			0.49			0.61			0.57	
Limestone, kg h^−1^	0.0			0.04			0.05			0.05	
Throughput, kg h^−1^ m^−2^	155			113			141			132	
Temperature of gasifier, °C	700			700			750			800	
Temperature of gasifying medium, °C	160			160			160			160	
Equivalence ratio, ER (−)	0.18	0.22	0.30	0.29	0.35	0.41	0.23	0.28	0.33	0.25	0.30
Air flow rate, dm^3^ min^−1^	6	7.2	10	7	8.5	10	7	8.5	10	7	8.5
Nitrogen flow rate, dm^3^ min^−1^	6	4.8	2	5	3.5	2	5	3.5	2	5	3.5
Fluidising medium flow rate, dm^3^ min^−1^	12	12	12	12	12	12	12	12	12	12	12
Superficial gas velocity based on the total product gas yield, m s^−1^ (*T*_g_)	0.21	0.24	0.24	0.22	0.21	0.20	0.24	0.23	0.23	0.25	0.24

a Later in the manuscript these tests are also referred to as campaign ECN Pt.III.

### 2.3 Tar measurement methods

The detailed description of the SPA tar sampling method, extraction and chromatographic analysis of tar is provided elsewhere.^21^ Briefly, SPA cartridges were assembled by packing 500 mg of aminopropyl silica sorbent. The sampling volume was adjusted to 100 ml of dry product gas. For each experimental condition duplicate SPA samples were taken. After sampling the cartridges were shipped to the University of Limerick – Ireland where the tar compounds were extracted from the sorbent with 3 × 600 μl of dichloromethane. *tert*-Butylcyclohexane and 4-ethoxy phenol were added as internal standards to the extracted tar solutions. An Agilent 7890A GC coupled with a triple-axis MSD 5975C was used for identification of the most abundant tar compounds. A Thermo Scientific Trace 1310 GC with a flame ionisation detector (GC-FID) was used for tar quantification. Calibration curves naphthalene/*tert*-butylcyclohexane and phenol/4-ethoxy phenol were applied to quantify the aromatic and phenolic tar, respectively. The FID detector was deemed more accurate than the MSD for tar quantification based on a statistical comparison of both methods.^21^ Benzene and toluene concentrations were measured along with the other permanent gases using on-line μGC (Varian, CP-4900). Their yields are presented as an average of four successive measurements conducted at three-minute intervals.

Tar yields are expressed on a mass basis as g_tar_ kg_poultry litter (dry and ash free-d.a.f.)_^−1^ in order to eliminate any dilution effect of the product gas when slight deviations of biomass feed rate occur,^22^ or when the oxygen to nitrogen ratio is reduced to adjust for lower ER.^23^ Tar yields are presented graphically but are also tabulated in the ESI†. Tabulated yields presented as g_tar_ kg_poultry litter (d.a.f.)_^−1^ enable fundamental studies of tar with respect to the feedstock, while yields presented as g_tar_ m_dry product gas_^−3^ are more useful for developers and operators of downstream applications such as tar mitigation technologies or using the product gas in internal combustion engine.

Total tar in this paper refers to GC detectable tar sampled by SPA inclusive of tar compounds from styrene (*M* ≈ 104 g mol^−1^) to benz[*a*]anthracene (*M* ≈ 228 g mol^−1^) as well as benzene and toluene measured by on-line micro GC instrument. The reason why total tar does not include light tar compounds (*i.e.* so called BTEX – Benzene, Toluene, Ethylbenzene, Xylene) sampled by SPA is due to delay between sampling and extraction of the SPA cartridges. As reported previously by Horvat *et al.*^24^ a significant portion of the volatile compounds such as benzene, toluene and xylene are lost during transport resulting in their quantitative underestimation as well as poor measurement repeatability. Instead, benzene and toluene measured by on-line μGC is combined with SPA sampled tar to sum up for total tar. Although, according to CEN/TS 15439, 2006 ^9^ benzene is excluded from the definition of tar, in the present work it is presented as a tar compound since its aromatic chemical structure is more characteristic of tar species than permanent gases. The results of poultry litter tar sampled by SPA are presented in duplicate for each gasification condition to show the repeatability of the measurements and the random errors associated with fluctuations in the feeding rate. It is evident from Table 3 that the measurement repeatability in this study is mediocre. One possible reason could be the relatively low tar content in the product gas (*i.e.* under 10 g_tar_ kg_poultry litter (d.a.f.)_^−1^ summing up compounds from styrene to benz[*a*]anthracene) along with previously mentioned losses during the analysis delay. Table 3 compares repeatability from three experimental campaigns conducted in 2012 (ECN Pt.I), 2013 (ECN Pt.II) and 2015 (ECN Pt.III-current study) using the same test rig but different feedstock as well as different post sampling treatment of the SPA samples. SPA tar samples from campaigns ECN Pt.I and ECN Pt.II were extracted on-site immediately after the sampling, while as mentioned previously, there was a delay before the extraction step of ECN Pt.III samples. SPA tar yields measured during experimental campaigns ECN Pt.I and ECN Pt.II were significantly higher^25^ compared with those in the current study. Repeatability was estimated using an open access excel spreadsheet^26^ model implementing calculations similar to those in the TAPPI standard T 1200, “Interlaboratory Evaluation of Test Methods to Determine TAPPI Repeatability and Reproducibility”.^27^ In short, coefficient of variance is calculated for SPA replicates of each experimental condition. So derived coefficients of variance are then combined into average covariance which enables expression of repeatability in percentage. Higher values in Table 3 indicate poor measurement agreement between replicates.

**Table 3:** Measurement repeatability of total SPA tar, phenol and naphthalene calculated for three experimental campaigns^a^

	Total SPA tar	Phenol	Naphthalene	Ref.
ECN Pt.I	9	11	15	Horvat *et al.*^25^
ECN Pt.II	13	11	9	Horvat *et al.*^25^
ECN Pt.III	68	66	71	Current study

a Repeatability is expressed in (%).

### 2.4 Limestone/lime characterisation methods

Limestone samples were analysed by the thermal gravimetric analyser (TGA). The elemental composition of limestone (C, H, N and S) was determined by a Vario EL cube elemental analyser. To reduce the uncertainties involved in measurements, elemental analyses were performed in triplicates. Results are presented on as received basis. Prior to analysis limestone grains were manually separated from the bed content (*i.e.* char, ash and silica sand). PerkinElmer's Pyris 1 TGA was employed to test the degree of calcination of the limestone. Approximately 20 mg of limestone grain was placed into an alumina pan without a lid and was heated from 30 to 995 °C at the rate of 20 °C min^−1^ under a nitrogen purge flowrate of 20 cm^3^ min^−1^. The same temperature program was performed under a carbon dioxide purge flowrate of 20 cm^3^ min^−1^.

## 3. Results and discussion

**Fig. 1:**
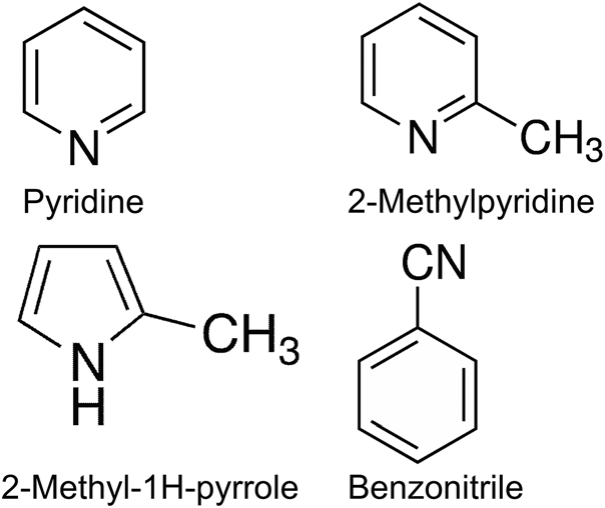
Nitrogen-containing compounds found in poultry litter tar.

The identified tar compounds are presented in Table 4 in the order in which they eluted. The composition of tar from poultry litter gasification is distinctively different from tar composition from conventional lignocellulosic fuels, specifically in terms of nitrogen-containing hydrocarbons. Most of the nitrogen in the poultry litter derives from the animal feed, excreta and feathers rather than from the bedding material. This nitrogen is chemically incorporated into protein molecules and urea.^28,29^ It is believed that the presence of significant amounts of pyridine, 2-methylpyridine, 2-methyl-1*H*-pyrrole and benzonitrile in the tar is due to the high level of nitrogen in the fuel (poultry litter). However, the question remains as to whether nitrogen-containing hydrocarbons arising from proteins retain their monomer structure or derive from reforming reactions between permanent gases (*i.e.* NO_*x*_, NH_3_, HCN) and the condensable fraction (*i.e.* tar) in the product gas. Nitrogen-containing compounds are normally not reported in the relevant gasification literature since insignificant amounts are generated from conventional lignocellulosic feedstock. Fig. 1 shows the structural formulae of the nitrogen-containing compounds identified in this study.

**Table 4:** Identified tar compounds with the retention time and classification according to Milne *et al.*^33^

Tar compound	Retention time (min)	Tar group
Benzene	4.65	Secondary
Pyridine*	7.15	Secondary
Toluene	7.90	Secondary
2-Methylpyridine	8.25	Secondary
2-Methyl-1*H*-pyrrole	9.81	Secondary
Ethylbenzene	11.38	Secondary
*p*-Xylene	11.68	Secondary
Styrene	12.49	Secondary
Benzonitrile*	15.85	Secondary
Phenol*	16.15	Secondary
Indene*	17.81	Secondary
*o*/*m*/*p*-Cresol*	18.25	Secondary
*o*/*m*/*p*-Cresol*	18.92	Secondary
1,2-Dihydronaphthalene	21.10	Secondary
Naphthalene*	22.18	Tertiary-PAH
Acenaphthylene*	29.36	Tertiary-PAH
2,4*A*-Dihydrofluorene	32.14	Secondary
Fluorene	32.57	Tertiary-PAH
Phenanthrene*	36.80	Tertiary-PAH
1-Methylphenanthrene	38.84	Tertiary-alkyl
4-Methylphenanthrene	39.22	Tertiary-alkyl
Pyrene	41.48	Tertiary-PAH
11*H*-Benzo[*b*]fluorene	41.86	Tertiary-PAH
Benzo[*a*]anthracene	45.85	Tertiary-PAH

The formation of nitrogen-containing hydrocarbons in the pyrolysis process has been investigated by Dignac *et al.*^30^ In the pyrolysates from fresh vegetables pyridine, pyrrole, benzonitrile and indole derivatives were detected among the other nitrogen-containing hydrocarbons. The authors attributed the pyridine derivatives to the pyrolysis of alanine-containing proteins and peptides, with the benzonitrile derivatives probably formed from pyrolysis of phenylalanine-containing proteins. Pyrrole and its derivatives were formed by cyclisation during pyrolysis of proteins containing the amino acids proline, hydroxyproline, glycine and glutamic acid, but could also be pyrolysis products of pigments such as chlorophyll. The proteins in poultry litter originate from waste feed and feathers, while the chlorophyll derivatives originate from bedding material and waste feed. Poultry excreta also contains nitrogen that possibly plays a role in the formation of nitrogen-containing hydrocarbons as indicated by Inoue *et al.*^31^ who analysed the products of liquefaction of ammonia and cellulose. Brebu and Spiridon^32^ investigated the thermal degradation of sheep wool, human hair and chicken feathers containing keratin proteins and attributed the formation of aromatic pyrroles and pyridines to the amino acids in the protein of keratin. The majority of the nitrogen-containing hydrocarbons were found in the aqueous phase of the pyrolysis condensate which needs to be taken into account in the development of tar cleaning and waste water treatment technologies.

**Fig. 2:**
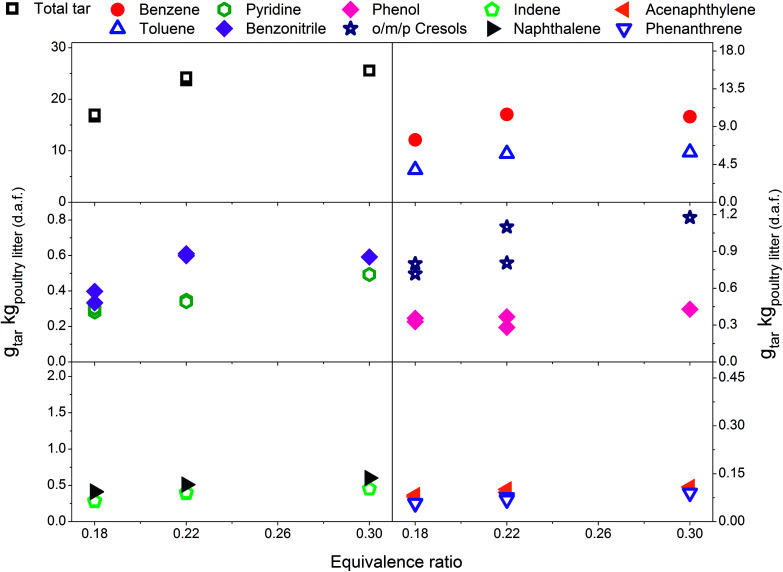
Equivalence ratio profile for the tar yields at reactor temperature of 700 °C without limestone addition.

**Fig. 3:**
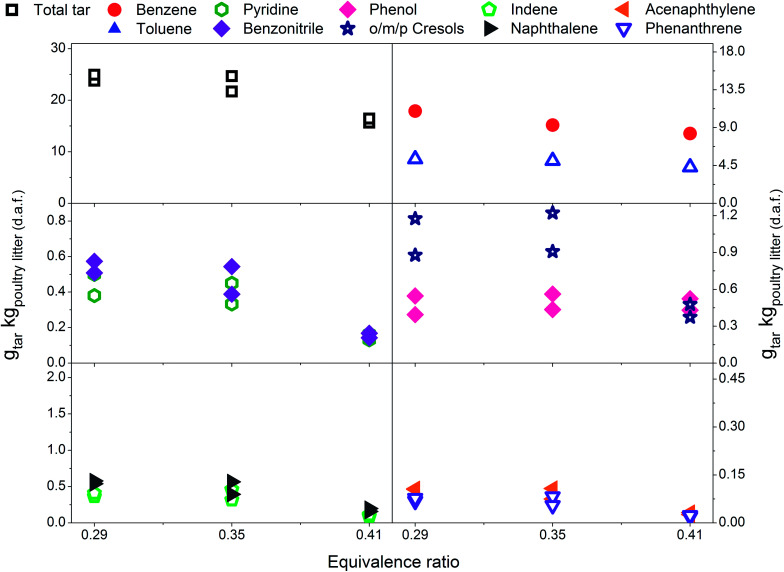
Equivalence ratio profile for the tar yields at reactor temperature of 700 °C with limestone addition.

**Fig. 4:**
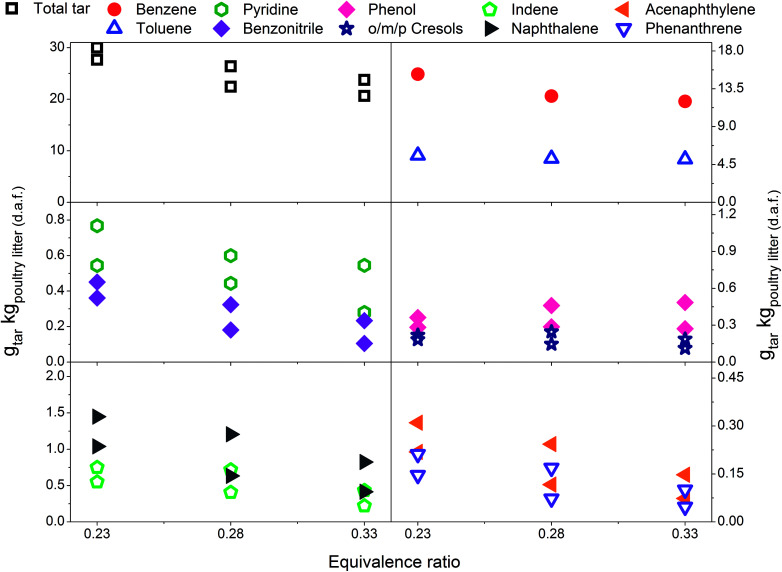
Equivalence ratio profile for the tar yields at reactor temperature of 750 °C with limestone addition.

**Fig. 5:**
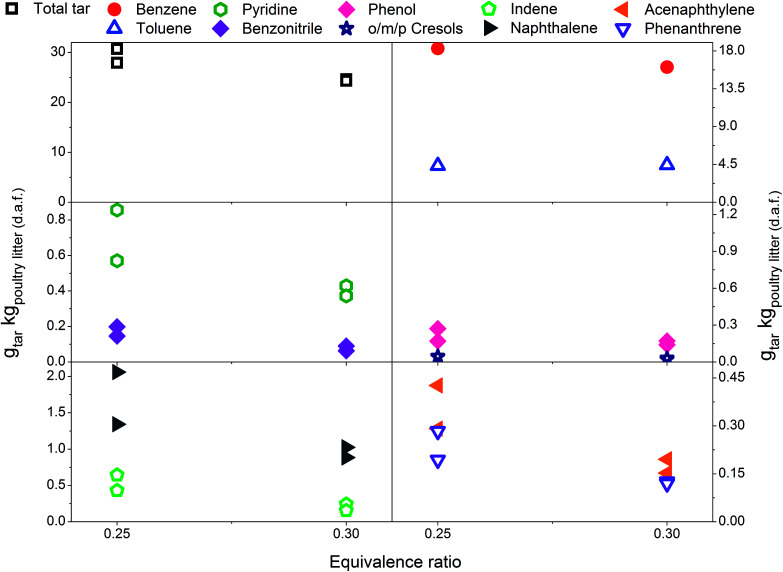
Equivalence ratio profile for the tar yields at reactor temperature of 800 °C with limestone addition.

**Fig. 6:**
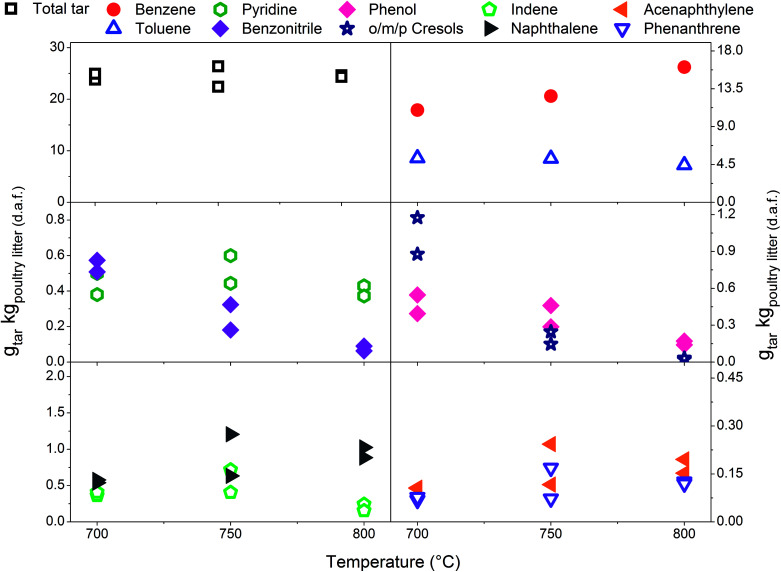
Temperature profile for the tar yields at an equivalence ratio of 0.29 ± 0.01 with limestone addition.

Eight individual tar compounds sampled by the SPA method (designated by * in Table 4) are presented quantitatively in Fig. 2–6. Pyridine and benzonitrile represent nitrogen-containing hydrocarbons while phenolic hydrocarbons mainly contain phenol and cresols. It should be noted that two isomers of cresol are summed and presented as a single quantity. Indene, naphthalene, acenaphthylene and phenanthrene are representatives of PAHs.

Table 5 includes the yields of principal permanent gases expressed in g_gas_ kg_poultry litter (d.a.f.)_^−1^. Although the yields of permanent gases are not the main focus of the present work, these data aim to support discussion on tar yield and composition.

**Table 5:** Yields^a^ of permanent gases during fluidised bed gasification of poultry litter

Test number	1	2	3	5	6	7	9	10	11	13	14
Feedstock type	Poultry litter			Poultry litter with 8% w/w limestone			Poultry litter with 8% w/w limestone			Poultry litter with 8% w/w limestone	
Temperature of gasifier, °C	700			700			750			800	
Equivalence ratio, ER (−)	0.18	0.22	0.30	0.29	0.35	0.41	0.23	0.28	0.33	0.25	0.30
Permanent gas yield, g_gas_ kg_poultry litter (d.a.f.)_^−1^											
H_2_	14.2	25.5	26.4	15.7	13.9	6.2	24.3	21.7	20.5	27.8	22.2
CH_4_	28.7	44.5	42.9	39.6	34.7	29.1	50.8	43.1	41.6	53.6	45.5
CO	145.5	259.5	294.5	191.1	187.4	150.2	274.9	264.3	255.6	336.2	258.2
CO_2_	483.2	636.7	749.2	733.1	813.1	844.8	656.8	687.3	762.7	743.6	770.7
C_2_H_4_	24.4	35.4	34.6	35.0	31.5	29.5	47.6	42.4	40.8	52.9	44.8
C_2_H_6_	5.6	9.5	9.6	7.5	7.0	5.3	6.6	6.2	6.1	4.1	3.7
C_2_H_2_	0.4	0.5	0.4	0.6	0.6	0.6	0.8	0.8	0.6	0.8	0.5
H_2_S	1.5	2.3	2.1	1.9	2.4	1.1	0.9	1.2	1.1	0.8	1.0
Sum	703.7	1014.0	1159.8	1024.6	1090.6	1067.0	1062.7	1067.0	1129.0	1219.8	1146.4

a Values calculated based on data from ref. 6.

The scale on the *y*-axis is kept the same in all graphs in order to simplify comparison of tar yields. The results indicate that tar yields from poultry litter gasification are lower than from feedstocks with a higher organic fraction. This was corroborated by only mild coloration of the white aminopropyl silica sorbent which typically turns dark yellow when product gas with high tar content is sampled. Low total tar yields can be attributed to the very specific composition of poultry litter which has a high ash content and low organic fraction, in particular low lignin content (Table 1). Lignin is known to be a tar precursor giving rise to higher total GC detectable tar and PAHs than cellulose and hemicellulose.^34,35^ However, smaller quantities of phenols and PAHs can also be formed from cellulose and hemicellulose.^36^ An ash content of 17.55 wt% (dry basis) in poultry litter is regarded as high but its composition and in particular the concentration of elements such as Ca, Mg, Al, Fe, Zn, Mn^6^ which exhibit catalytic tar reduction activity could have played a role in the total tar reduction.^37^ The total tar yields presented in Fig. 2–6 (15.7 to 30.7 g_total tar_ kg_poultry litter (d.a.f.)_^−1^) include benzene and toluene measured by on-line micro GC. It is worth mentioning that if benzene and toluene yields measured by on-line micro GC are subtracted from the total tar from poultry litter, the yield of total tar would drop to between 3.1 to 10.3 g_total tar_ kg_poultry litter (d.a.f.)_^−1^. It has been reported previously^24^ that on-line μGC measurements give considerable higher benzene and toluene quantities comparing to SPA. Additionally Brage *et al.*^38^ showed that SPA sampling is far superior for quantification of BTEX compounds compared to traditional cold trapping. Therefore, comparing the total tar yield from the relevant literature is complicated due to differences in defining tar, sampling conditions, analytical instrument calibration and reported units. Kinoshita *et al.*^23^ reported total tar yields in the range of 40–45 g_total tar_ kg_dry wood sawdust_^−1^ while conducting the tests under similar ER conditions to those reported here. The tar sampling set up employed was a combination of dry and wet cold trapping Horvat *et al.*^25^ measured total tar between 14–34 g_total tar_ kg_biomass (d.a.f.)_^−1^ from raw and torrefied *Miscanthus x giganteus* respectively using the same experimental reactor as being used for this study. In that case tar compounds in the molecular weight range from benzene to benzo[*k*]fluoranthene were sampled by means of the SPA method. Compared to the poultry litter, both raw and torrefied *Miscanthus x giganteus* have lower ash contents of 2.8 and 4.2 wt% and higher lignin content of 21 and 43 wt%, respectively.

### 3.1 Effect of equivalence ratio on tar yield and composition – without limestone addition

Fig. 2 includes total tar yields and composition over the ER range between 0.18 and 0.30 at 700 °C, without addition of the limestone to the poultry litter. It is evident that total tar as well as nitrogen and oxygen containing tar compounds slightly increase with the ER. Such an observation is in disagreement to the results presented by Kinoshita *et al.*^23^ and Hanping *et al.*^39^ employing wood sawdust, peanut shell and wheat straw as a fuel. They observed a decrease of total tar and oxygen containing tar compounds with increasing ER while keeping the temperature at 700 and 800 °C, respectively. However, more recently Horvat *et al.*^25^ found that at constant temperature, the ER has relatively little impact on the yield or composition of tar from a grassy biomass. These three studies^23,25,39^ have been considered for comparison due to their ability to study the effect of ER separately from the temperature effect on tar evolution. The concentration of PAH compounds in this study increases with increasing ER. The yields for benzene and toluene increase slightly and then level-off at an ER of 0.3. Similar to the total tar, the yield of product gases increases with ER. At higher ER more oxygen is available for char conversion into product gas as well as reacting with permanent gases and tar. Therefore, as expected an increase in product gas yield and carbon conversion (from 49.1% at ER of 0.18 to over 70% at ER of 0.22 and 0.3) was observed. However, this was accompanied by an increase in tar yield. Poultry litter comprise a very high fraction of extractives (40% on a dry basis, Table 1) but their exact composition is not known. Literature information on the extractive fraction in poultry litter is scarce. It is not known to what extend these extractives contribute to either the product gas or tar yields.

### 3.2 Effect of equivalence ratio on tar yield and composition-with limestone addition

Fig. 3 presents tar yields for the experiments undertaken between an ER of 0.29 and 0.41, at a gasification temperature of 700 °C using poultry litter blended with limestone (8% w/w). Fig. 2 and 3 show data for the same temperature, but the ERs correspond to two different ranges (0.18–0.30 *vs.* 0.29–0.41). Differences in the ER derive from daily variations of fuel feeding rate, despite efforts being made to maintain a constant feeding rate throughout the experimental campaign. Due to the agglomeration issue associated with the raw poultry litter, it was decided to add limestone (CaCO_3_), however no adjustment was made to the calibration of the feeding system. Since the range of ER differs for both the limestone blended and raw poultry litter, it is not possible to draw unambiguous conclusions regarding whether the difference in tar yields is due solely to the effect of limestone. The only gasification conditions allowing direct evaluation of the effect of limestone are ER 0.30; *T* 700 °C; without limestone addition and ER 0.29; *T* 700 °C; with limestone addition, respectively. There was 5% less of total tar from the test without limestone addition. The difference between the yields of individual tar compounds vary between – 9 and 21.3% taking the test without limestone addition as a reference value. These findings show no significant effect of limestone addition on the tar yield and its composition at the lowest gasification temperature. Permanent gases measurements actually reveal higher yields of H_2_, CH_4_, CO in the test without limestone addition, while the concentration of CO_2_ was similar for both scenarios. Correlation exist between the yields of permanent gases and carbon conversion efficiency which appears to be 81.8% in the test without limestone addition and 70.8% in the test with limestone addition. Although it has been found that alkaline earth metal oxides (CaO/MgO) employed in steam gasification increase the yields of permanent gases (H_2_, CO_2_) by promoting the decomposition reactions of tar and light hydrocarbon^13,40,41^ it seems this phenomena did not occur in gasification test at 700 °C. There could be two potential reasons for low catalytic activity of limestone: limited calcination at low temperature and adsorption of sulphur. In order to understand the transformation of limestone during gasification tests additional characterisation of limestone grains separated from the bed after tests was carried out. These results are discussed in detail in Section 3.4 and they show that at 700 °C limestone was only partially calcined, therefore inactive towards tar reduction. According to Florin and Harris^42^ calcination of limestone forming catalytically active lime does take place in an inert atmosphere at 700 °C. In an atmosphere containing CO_2_ the carbonation/calcination reactions shift to higher temperature comparing to inert atmosphere because of reversible nature of the calcination reaction at an equilibrium condition.^17^ Lime alteration can also occur in the presence of gaseous H_2_S (Table 5) forming CaS.^43^ Direct evidence of catalytic deactivation of lime by H_2_S has not been found in the literature, but catalyst poisoning caused by sulphur has been reported previously.^44,45^ The sulphur content in the recovered limestone/lime after the gasification at 700 °C increased 3 times compared to the unused limestone (Table 6) proving chemical interaction between H_2_S and the limestone/lime.

**Table 6:** Elemental composition of limestone/lime samples expressed in wt% on as received basis

	Unused limestone	Limestone 700 °C	Limestone 750 °C	Limestone 800 °C
N	0.004	0.003	0.068	0.001
	0.001	0.006	0.009	0
	0.005	0.005	0.007	0.001
C	16.110	14.551	0.327	0.796
	16.182	14.394	4.451	4.064
	16.102	14.100	4.908	5.433
H	0.040	0.121	0.338	1.047
	0.048	0.120	0.210	1.194
	0.037	0.113	0.433	1.257
S	0.119	0.456	1.562	1.860
	0.142	0.444	1.731	1.226
	0.122	0.442	2.066	0.669

In Fig. 3 a reduction in total tar is observed over the tested ER range when poultry litter was blended with the limestone. Similar trends are observed in Fig. 4 and 5 showing decreasing total tar over the range of ER tested at gasification temperatures of 750 and 800 °C, respectively. It is worth emphasizing that the total tar and yields of individual tar species show the same trend. Benzene and toluene also follow a decreasing trend as the other SPA sampled tar compounds do, although toluene does not seem to be notably affected over the ER range tested. There are at least two factors which could have caused decrease of tar: higher content of reactive oxygen (higher ER) and presence of limestone whose calcination degree increased with temperature. From the data available in Table 5 an increase in ER results in a reduction of both H_2_ and CO concentration and an increase in CO_2_ in the product gas due to combustion of the volatiles and char. Despite its higher concentration, it seems that the CO_2_ did not impact on the catalytic ability of the lime due to carbonization into limestone over the timeframe of the experiments at 750 and 800 °C. Thermal gravimetric analysis of limestone/lime after gasification revealed that the degree of *in situ* calcination was significantly higher at 750 and 800 °C. Moreover, over 10-folds higher sulphur content was observed in the recovered limestone/lime from the gasification experiments compared to the unused limestone. Despite high sulphur adsorption, no clear evidence of reduced catalytic tar reduction capacity of limestone/lime was found during the gasification.

Delgado *et al.*^40^ and Simell *et al.*^12^ reported rapid catalytic deactivation of limestone/lime as a result of coke deposition on the surface of active sites. The authors also stated that both wet (steam) and dry (CO_2_) gasification eliminate coke from the surface which could explain the increased catalytic activity with increasing ER. Wet and dry gasification reactions are strongly endothermic. Therefore, they are more likely to play a crucial role in the tests at 750 and 800 °C than at 700 °C. Moreover, at higher ER more oxygen is available to oxidise any deposited coke. It is not clear how the oxygen itself affects the redox equilibrium of limestone/lime. Of relevance to the present work, Campoy *et al.*^46^ conducted gasification tests in an air blown bubbling fluidised bed reactor using wood pellets as a fuel. Ofite, a silicate subvolcanic rock was compared to calcined limestone over an ER range between 0.23 and 0.36. The yields of gravimetric tar decreased only at an ER above 0.3 while employing silicate rock. On the other hand, the addition of calcined limestone resulted in a slight decrease of gravimetric tar over the entire ER range tested. However, notable variations have been found between silicate rock tests and the tests with added calcined limestone. Gravimetric tar quantities between 40 and 50 g_tar_ kg_wood (d.a.f.)_^−1^ resulted from silicate rock, while between 25 and 30 g_tar_ kg_wood (d.a.f.)_^−1^ were measured after the addition of calcined limestone.

In a nutshell, there are indications that catalytic tar reduction took place when the gasification tests were performed at 750 and 800 °C. However, tar reduction cannot be unambiguously attributed to the calcined limestone/lime because oxygen content, ER, could play a crucial role in tar reduction as well.

### 3.3 Effect of temperature on tar yield and composition-with limestone addition

In Fig. 2–4 the yields of phenols (from 0.11 to 1.22 g_tar_ kg_poultry litter (d.a.f.)_^−1^) and benzonitrile (from 0.10 to 0.61 g_tar_ kg_poultry litter (d.a.f.)_^−1^) are relatively high at low gasification temperatures between 700 and 750 °C. However, at 800 °C only between 0.02 and 0.27 g_tar_ kg_poultry litter (d.a.f.)_^−1^, of phenols and benzonitrile are observed due to their conversion *via* demethylation, dehydration^47^ and denitrification reactions.^48^ Reforming mechanisms using model compounds such as pyridine, pyrrole and indole have been studied in the context of thermochemical conversion of coal.^48,49^ Liu *et al.*^48^ measured NH_3_ and HCN as the main gaseous products from conversion of nitrogen-containing hydrocarbons. Gasification of indole was carried out in supercritical water and the authors concluded that one portion of indole converted directly into aromatic compounds without nitrogen by releasing ammonia, while another portion of indole was converted into nitrogen-containing aromatic compounds such as aniline, *o*-toluidine and 9-nitroso-9*H*-carbazole. Zhao *et al.*^49^ pyrolysed pyridine and pyrrole at 600–1200 °C in a flow reactor. H_2_ and HCN were measured in order to determine the thermal stability of pyridine and pyrrole. The results showed that the thermal stability of pyridine is greater since significant production of HCN was observed at 825 °C while pyrrole generated notable amounts of HCN at 775 °C. A thermal degradation (*i.e.* ring-opening) mechanism was proposed for both nitrogen-containing hydrocarbons studied. The pyridine ring undergoes a series of free radical reactions resulting in H_2_ and an aliphatic ·R–CN. On the other hand, it is assumed that pyrrole undergoes direct ring opening, therefore reforming into an aliphatic R–CN without passing through free radical reactions.

Fig. 6 presents the total tar yields and quantities of ten individual tar species with respect to gasification temperature at an ER of 0.29 ± 0.01. The yield of total tar over the temperature range tested remains steady which is considered as an atypical observation with respect to earlier literature findings. Depending on the range of temperature tested and the tar sampling method employed the yields of total tar either decreased^22,50,51^ or exhibited the peak yield at around 750 °C followed by a decrease.^25,33,52^ Steady total tar yields are attributed to the prevailing effect of high benzene concentrations which increase with temperature over less abundant and diminishing species such as benzonitrile, phenols and indene. It should be noted that if benzene and toluene are excluded, the total SPA tar summed from styrene to benz[*a*]anthracene shows a significant reduction with increasing temperature.

According to Delgado *et al.*^40^ higher reaction temperature favours catalytic activity of lime for tar destruction in the temperature range of 780–880 °C in a fluidised bed biomass gasifier. Although, the authors also observed catalyst deactivation due to coke formation and adsorption on the active sites regeneration of lime by coke removal was effectively achieved by steam and dry (CO_2_) gasification. Fig. 6 indicates that catalytic activity of limestone/lime promoted by elevated temperature could have reduced heterocyclic tar (*i.e.* phenols, benzonitrile), toluene and indene, while no reduction effect is observed for benzene and PAHs. Similar quantitative curves of individual tar compounds presented in Fig. 6 were previously attributed solely to the temperature effect.^23,25^ Saw and Pang^13^ investigated the influence of lime loading on the tar yields in a dual fluidised bed steam gasifier operated at 710–750 °C. They observed catalytically driven reduction of tar including tar species in classes from C2 to C5 according to ECN classification.^52^ However, benzene was not included in their study. Simell *et al.*^12^ tested calcined carbonated rock for its catalytic reforming potency using benzene, toluene and naphthalene as model tar compounds. The order of reforming toluene > naphthalene ≫ benzene indicated greater chemical stability of benzene for catalytic reforming at 900 °C.

In summary, perhaps there is a trade-off between phenomena including limestone calcination and lime carbonisation,^12,17^ coke deposition, coke gasification (*i.e.* coke removal),^12,40^ and sulphur poisoning.^44,45^ influencing its catalytic activity. In any case, in the present work there is no incontrovertible evidence of limestone/lime catalytic activity with respect to temperature and associated tar reduction.

Indene has its peak production at 750 °C while the PAH yields gradually increases with temperature. The nitrogen-containing hydrocarbons show different behaviour with respect to increasing temperature. Benzonitrile yield decreases while that of pyridine remains relatively high at elevated temperatures indicating its high thermal stability. Pyridine has a non-branching aromatic chemical structure while the benzonitrile substituent makes it more thermally sensitive. This observation was confirmed by Zhao *et al.*^49^ who reported that pyridine undergoes thermal degradation at temperatures above 825 °C. Thermal decomposition of nitrogen-containing tar compounds suggests higher yields of NH_3_ which was monitored throughout experimental campaign,^6^ but due to the rapid thermal decomposition of NH_3_ its concentration decreased sharply with the temperature.^53^ At this point it is worth mentioning that NO_*x*_ emissions might be elevated upon combustion of the N-containing product gas. An increase in benzene yield with the temperature correlates with reforming of compounds such as phenols, toluene and benzonitrile.^47^

### 3.4 Limestone/lime properties before and after gasification

**Fig. 7:**
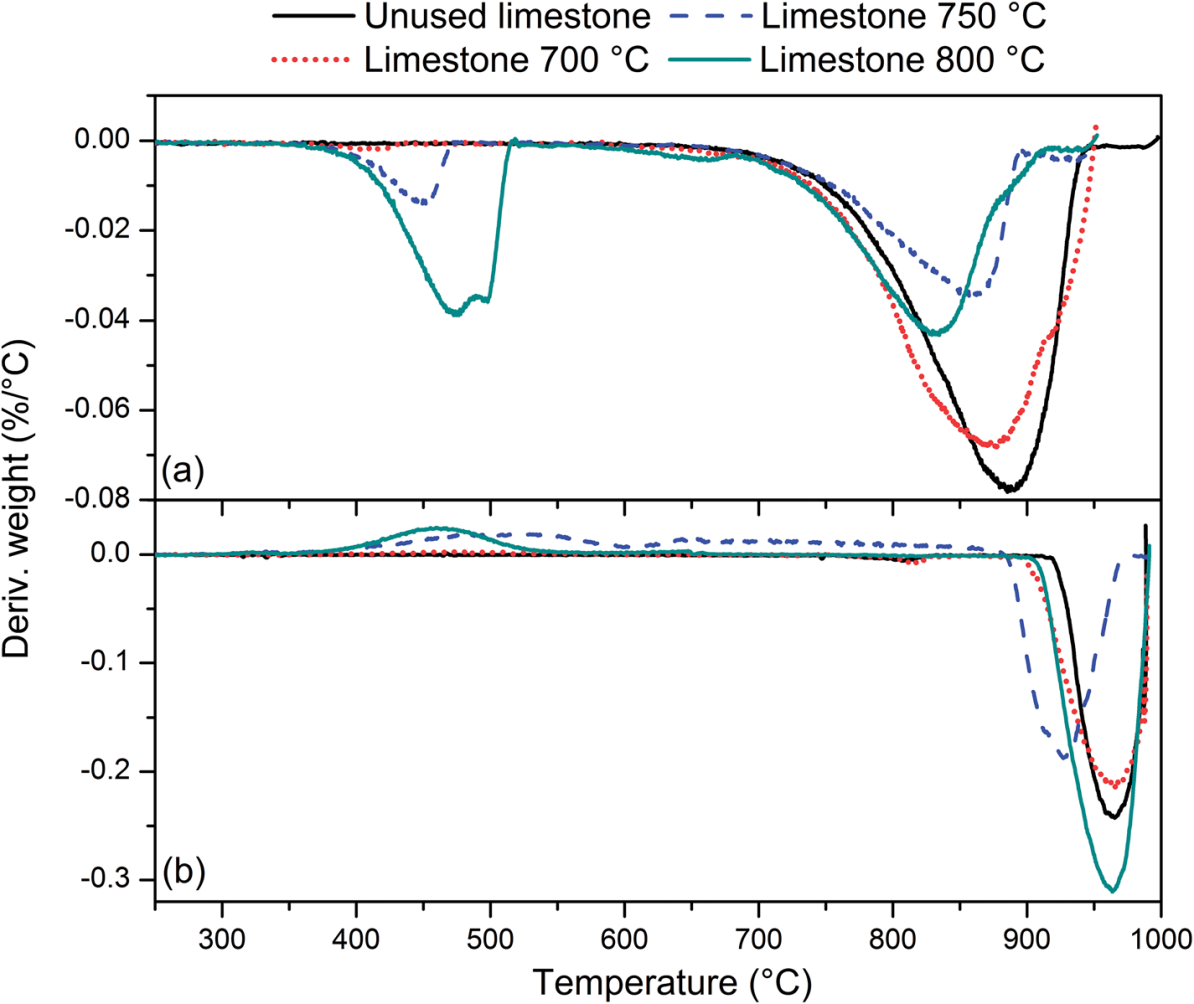
Differential thermo-gravimetric (DTG) profiles for limestone/lime samples in (a) nitrogen and (b) carbon dioxide atmosphere.

In order to get better insight into limestone/lime transformation during poultry litter gasification additional tests were performed. Fig. 7 presents differential thermo-gravimetric (DTG) profiles of unused limestone together with the limestone/lime used in the gasification tests at 700, 750 and 800 °C, respectively. Profiles (Fig. 7a) correspond to inert nitrogen atmosphere, while the profiles (Fig. 7b) refer to reactive carbon dioxide atmosphere. DTG profile of unused limestone in nitrogen does not show the presence of Ca(OH)_2_ which would dissociate into CaO and H_2_O. The only peak that was observed at temperature between 750–950 °C is associated to the calcination reaction CaCO_3_ = CaO + CO_2_.^17^ Limestone/lime samples taken out from gasification reactor show two decomposition regions. The first peak between 370 and 520 °C increases with the gasification temperature (from about 1 to 13% of mass loss). This can be associated to chemical reaction of hydroscopic CaO with the steam^17^ and H_2_S.^19,54^ The second peak between 750–950 °C decreases with the gasification temperature indicating temperature driven conversion of CaCO_3_ into CaO. The DTG profiles from nitrogen atmosphere demonstrate that limestone from 700 °C gasification did not reach notable degree of calcination. The calcination of unused limestone was assumed to be completed when mass reduction of 45.5% was achieved due to CO_2_ desorption in nitrogen atmosphere. The difference between the mass loss from samples after gasification and the mass loss from unused limestone shows a degree of calcination at different temperatures. Smaller is the peak at temperatures between 750–950 °C (Fig. 7a), higher is the degree of *in situ* calcination during gasification. The mass loss of limestone/lime during *in situ* gasification was about 2% (700 °C), 28% (750 °C) and 24% (800 °C). The DTG profiles of carbon dioxide are in agreement with observations above. Unused limestone and limestone from 700 °C gasification did not uptake CO_2_ since CaCO_3_ was predominant chemical form. The region between 900 and 990 °C denotes decomposition of CaCO_3_ into CaO and CO_2_.^17^ In contrast, limestone form 750 and 800 °C gasification adsorbed CO_2_ in the region between 350 and 800 °C. According to Bedyk *et al.*^17^ in this region Ca(OH)_2_ and CaO react with CO_2_ giving CaCO_3_ which is again calcined at temperature above 830 °C.

The elemental composition of limestone/lime samples presented in Table 6 reveals that carbon content dropped, while hydrogen and sulphur increased with the gasification temperature. This again is the evidence that CO_2_ was released during gasification. At the higher temperature the amount of carbon retained was low. Along with that increasing hydrogen content indicates the presence of Ca(OH)_2_ formed from hygroscopic CaO when in contact with steam. Increased sulphur content could be a proof of CaS in the limestone/lime from 700, 750 and 800 °C gasification. In combustion atmosphere CaS decomposes into CaO and SO_2_.^55^

Based on a characterisation tests it can be concluded that the recovered limestone/lime from 750 and 800 °C gasification was calcined *in situ* to some degree, while this cannot be confirmed for the limestone obtained from 700 °C gasification test. A fraction of CaO reacted with a gaseous H_2_S forming CaS. Therefore, the catalytic capacity for tar reduction is probably a trade-off between calcinated limestone (*i.e.* CaO) and CaS.

## 4. Conclusions

Yields and composition of tar from the bubbling fluidised bed gasification of poultry litter were investigated as a function of temperature, equivalence ratio (ER) and limestone addition to the feedstock. Principally, limestone was added in order to reduce the risk of bed agglomeration. Along with that its capacity for catalytic tar reduction has been investigated. For the range of gasification conditions tested, the following conclusions can be drawn: (1) due to the high content of catalytically active inorganic species and low lignin content, poultry litter generates low yields of total tar (*i.e.* sum of SPA tar + benzene and toluene measured by micro GC) in the range from 15.7 to 30.7 g_tar_ kg_poultry litter (d.a.f.)_^−1^ for the tested temperatures between 700 to 800 °C. At this point it needs to be noted that on-line micro GC measures considerable higher benzene and toluene quantities compared to more commonly employed off-line techniques. (2) The composition of tar from poultry litter gasification is remarkably different from those of conventional lignocellulosic biomass. Nitrogen incorporated in the protein structures of animal feed, excreta and feathers is likely the reason for the significant amounts of nitrogen containing hydrocarbons detected in tar (3) limestone addition to the poultry litter does not result in a tar reduction effect based on comparing two tests at an ER of 0.30 ± 0.1 and at a temperature 700 °C. (4) *In situ* calcination of limestone does not occur at 700 °C, but it does occur at 750 °C and 800 °C gasification. Limestone/lime adsorbs sulphur contaminant with the increasing temperature. (5) Temperature is an effective measure to reduce heterocyclic tar compounds such as toluene and indene but the amount of PAHs and benzene increases. Atypically constant total tar yield over the temperature range tested is attributed to the prevailing effect of increasing benzene yield with temperature. There is no incontrovertible evidence of limestone/lime catalytic activity with respect to temperature and associated tar reduction. (6) The measurement campaign once again revealed the issue regarding uncertainty of tar data due to the differences in tar definition, sampling conditions, analytical instrumentation and reported units across the scientific community. (7) The ER shows a distinctive effect on tar yield. In the absence of limestone tar yields increase, while the opposite trend was observed in the presence of limestone. However, tar reduction cannot be unambiguously attributed to calcined limestone/lime as ER may play crucial role in tar reduction as well.

## Conflicts of interest

There are no conflicts to declare.

## Supplementary Material

RA-009-C9RA02548K-s001
