# Supplementary material for: Tar yield and composition from poultry litter gasification in a fluidised bed reactor: effects of equivalence ratio, temperature and limestone addition
Source: RSC Adv. 2019 Apr 30;9(23):13283–96. doi: 10.1039/c9ra02548k (PMC9063797; doi:10.1039/c9ra02548k)
Supplement: RA-009-C9RA02548K-s001 [file RA-009-C9RA02548K-s001.pdf]

Electronic Supplementary Material (ESI) for RSC Advances.
This journal is © The Royal Society of Chemistry 2019

SUPPORTING INFORMATION

Table S1: Tabulated tar yields expressed as g_{tar} $kg^{-1}_{poultry\ litter\ (d.a.f.)}$

						Poultry litter							
Process conditions	Duplicate measurement	Benzene†	Pyridine*	Toluene†	Benzonitrile*	Phenol*	Indene*	o/m/p-Cresol*	Naphthalene*	Acenaphthylene*	Phenanthrene*	Total tar SPA	Total tar SPA + micro GC
700 °C/0.18	1	7.43	0.282	3.87	0.334	0.328	0.275	0.717	0.407	0.078	0.057	5.346	16.646
	2		0.303		0.398	0.356	0.286	0.800	0.416	0.084	0.058	5.706	17.006
700 °C/0.22	1	10.46	0.348	5.78	0.610	0.282	0.387	0.806	0.509	0.101	0.076	7.454	23.694
	2		0.340		0.599	0.368	0.399	1.099	0.512	0.092	0.069	7.978	24.218
700 °C/0.30	1	10.19	0.493	5.95	0.592	0.430	0.452	1.176	0.601	0.109	0.090	9.339	25.579
	2		/		/	/	/	/	/	/	/	/	/
						Poultry litter with 8 % w/w limestone							
700 °C/0.29	1	10.97	0.380	5.26	0.508	0.394	0.362	0.878	0.540	0.105	0.068	7.569	23.799
	2		0.500		0.573	0.547	0.407	1.175	0.578	0.107	0.077	8.702	24.932
700 °C/0.35	1	9.30	0.450	5.07	0.544	0.561	0.459	1.221	0.566	0.108	0.082	10.294	24.664
	2		0.333		0.389	0.436	0.316	0.907	0.393	0.075	0.055	7.310	21.680
700 °C/0.41	1	8.30	0.131	4.30	0.142	0.431	0.090	0.373	0.159	0.026	0.019	3.050	15.650
	2		0.165		0.168	0.524	0.109	0.478	0.198	0.032	0.024	3.837	16.437
750 °C/0.23	1	15.24	0.768	5.58	0.451	0.363	0.749	0.218	1.449	0.310	0.212	9.209	30.029
	2		0.544		0.361	0.283	0.550	0.182	1.039	0.219	0.146	6.838	27.658
750 °C/0.28	1	12.63	0.444	5.20	0.181	0.287	0.406	0.146	0.634	0.117	0.073	4.595	22.425
	2		0.600		0.324	0.460	0.717	0.247	1.205	0.243	0.169	8.550	26.380
750 °C/0.33	1	12.00	0.545	5.12	0.233	0.485	0.432	0.186	0.825	0.147	0.100	6.628	23.748
	2		0.280		0.105	0.271	0.221	0.112	0.414	0.073	0.047	3.519	20.639
800 °C/0.25	1	18.30	0.571	4.35	0.146	0.171	0.430	0.041	1.344	0.291	0.193	5.290	27.940
	2		0.856		0.199	0.272	0.643	0.047	2.061	0.427	0.284	8.054	30.704
800 °C/0.30	1	16.08	0.429	4.44	0.090	0.172	0.243	0.021	1.025	0.196	0.126	4.117	24.637
	2		0.373		0.063	0.140	0.155	0.031	0.884	0.153	0.120	3.812	24.332

† measured by on-line micro GC instrument; * measured by off-line SPA method

/ Due to the damage of SPA sample during the shipment duplicate data concerning poultry litter 700 °C/0.30 is not available.

Table S2: Tabulated tar yields expressed as $g_{tar}\ m^{-3}{}_{dry\ product\ gas}$

Process conditions	Duplicate measurement	Benzene†	Pyridine*	Toluene†	Benzonitrile*	Phenol*	Indene*	o/m/p-Cresol*	Naphthalene*	Acenaphthylene*	Phenanthrene*	Total tar SPA	Total tar SPA + micro GC
Poultry litter													
700 °C/0.18	1	10.17	0.124	5.31	0.147	0.144	0.121	0.316	0.179	0.034	0.025	2.355	17.837
	2		0.133		0.175	0.157	0.126	0.352	0.183	0.037	0.026	2.514	17.995
700 °C/0.22	1	9.97	0.135	5.50	0.237	0.110	0.151	0.314	0.198	0.039	0.030	3.248	18.718
	2		0.132		0.233	0.143	0.155	0.428	0.199	0.036	0.027	3.104	18.573
700 °C/0.30	1	9.18	0.194	5.36	0.233	0.169	0.178	0.463	0.237	0.043	0.035	3.677	18.214
	2		/		/	/	/	/	/	/	/	/	/
Poultry litter with 8 % w/w limestone													
700 °C/0.29	1	10.12	0.120	4.86	0.160	0.124	0.114	0.277	0.170	0.033	0.022	2.388	17.361
	2		0.158		0.181	0.172	0.128	0.371	0.182	0.034	0.024	2.745	17.718
700 °C/0.35	1	8.48	0.144	4.62	0.174	0.180	0.147	0.391	0.181	0.034	0.026	3.299	16.405
	2		0.107		0.125	0.140	0.101	0.291	0.126	0.024	0.018	2.343	15.448
700 °C/0.41	1	8.41	0.045	4.35	0.048	0.146	0.030	0.126	0.054	0.009	0.006	1.034	13.799
	2		0.056		0.057	0.178	0.037	0.162	0.067	0.011	0.008	1.303	14.068
750 °C/0.23	1	13.74	0.277	5.03	0.163	0.131	0.270	0.079	0.523	0.112	0.076	3.324	22.101
	2		0.196		0.130	0.102	0.198	0.066	0.375	0.079	0.053	2.469	21.245
750 °C/0.28	1	11.91	0.166	4.91	0.068	0.107	0.151	0.054	0.237	0.044	0.027	1.715	18.534
	2		0.224		0.121	0.172	0.267	0.092	0.450	0.091	0.063	3.190	20.010
750 °C/0.33	1	11.11	0.206	4.74	0.088	0.184	0.164	0.070	0.312	0.056	0.038	2.511	18.360
	2		0.106		0.040	0.103	0.084	0.042	0.157	0.028	0.018	1.333	17.182
800 °C/0.25	1	13.65	0.182	3.24	0.047	0.054	0.137	0.013	0.429	0.093	0.062	1.690	18.588
	2		0.274		0.063	0.087	0.205	0.015	0.658	0.136	0.091	2.573	19.471
800 °C/0.30	1	13.40	0.146	3.70	0.031	0.059	0.083	0.007	0.350	0.067	0.043	1.405	18.498
	2		0.127		0.022	0.048	0.053	0.011	0.302	0.052	0.041	1.301	18.394

† measured by on-line micro GC instrument; * measured by off-line SPA method

/ Due to the damage of SPA samples during the shipment duplicate data concerning poultry litter 700 °C/0.30 is not available.
